# The Efficacy of Marijuana Use for Pain Relief in Adults With Sickle Cell Disease: A Systematic Review

**DOI:** 10.7759/cureus.24962

**Published:** 2022-05-13

**Authors:** Christian N Paulsingh, Mohamed B Mohamed, Mohamed S Elhaj, Nusyba Mohamed, Tarig H Ahmed, Trisha Singh, Zahir Mohammed, Safeera Khan

**Affiliations:** 1 Internal Medicine, California Institute of Behavioral Neurosciences & Psychology, Fairfield, USA

**Keywords:** pain relief, marijauna use in sickle cell disease, hemoglobin sickle cell, vaso-occlusive crisis, cannabis (marijuana), sickle cell crisis, sickle cell disease complications, sickle cell disease: scd

## Abstract

Sickle Cell Disease (SCD) is a disease that affects many around the world and often accounts for frequent hospital admissions every year, secondary to uncontrolled pain. Marijuana is increasingly being used for its medicinal ability to treat pain in chronic medical conditions. Therefore, it is imperative to determine how effective it would be in providing pain relief to patients with SCD. We systematically screened five databases for relevant data: PubMed, Medline, PubMed Central (PMC), Cochrane Library, and Google Scholar. The inclusion and exclusion criteria were implemented. A quality appraisal was then done using the Cochrane Bias assessment for randomized controlled trials (RCTs), Newcastle-Ottawa tool for observational studies, and Scale for the Assessment of Narrative Review Articles (SANRA) checklist for traditional review articles. From seven articles, information was gathered; one systematic review, one RCT, two surveys, one cross-sectional study, one retrospective study, and one questionnaire-based study. Our review concluded that based on the literature assessed, marijuana use in SCD patients either worsened their painful crises or offered little to no help compared to opioids or hydroxyurea usage. There were limited RCTs published in addition to papers investigating the long-term effects of marijuana use in SCD. We hope that further data is gathered in these areas to sufficiently address whether cannabis use is efficacious for pain relief in patients with SCD.

## Introduction and background

South America, Central America, Sub-Saharan Africa, the Caribbean, Saudi Arabia, Turkey, Italy, Greece, and India all share a common issue. While sickle cell disease (SCD) affects millions worldwide, if your ancestors came from one of these regions, the chances are that there is an increased probability of being affected by this disorder according to the Centers for Disease Control and Prevention [1].

SCD results in more than 113,000 hospital admissions a year, most for uncontrolled pain, and currently affects more than 100,000 adults in the United States [2]. SCD represents a group of diseases with autosomal recessive inheritance, which occurs after a point mutation in the β chain of hemoglobin (substitution of a single amino acid in the β chain of hemoglobin) and leads to the formation of abnormal sickle hemoglobin (HbS) [3]. There are numerous types of SCD; the most prominent are sickle cell anemia, sickle hemoglobin-C disease (HbSC), sickle beta-plus thalassemia, and sickle beta-zero thalassemia [3]. Polymerization of HbS occurs as a result of deoxygenation of hemoglobin along with the creation of deformed sickle-shaped red blood cells (RBCs). The sickling of RBCs eventually results in permanent damage to the RBC cell membrane [3-5]. All these abnormal RBCs have a greater potential of developing complications such as hemolysis, chronic anemia, increased risk of inflammation, and finally vaso-occlusion [5]. SCD can lead to various symptoms and fallouts, namely sickle cell crisis, vaso-occlusive crisis (VOC) leading to pain, ischemia, necrosis, and finally organ damage, acute chest syndrome, and other complications. RBC breakdown and chronic anemia can lead to tissue hypoxia and multiple organ damage and, therefore, increase the risk of untimely demise [3-7].

SCD considerably reduces life expectancy by approximately 30 years [3]. The latest advancement in the management of SCD is voxelotor. This is a new original, orally administered drug that can modify the underlying disease pathology (by increasing the affinity between oxygen and Hb) and prevent the sickling of red blood cells [8]. Numerous case series and clinical trials have noted the benefits and safety of voxelotor therapy in SCD [8]. Pain in SCD is complex as people can have acute and chronic pain, neuropathic pain, and nociceptive pain, and can even lead to central and peripheral sensitization to pain [9]. Primarily, patients with SCD who are in pain are treated with opioid medications. However, this is often not sufficient and can lead to an increased risk for abuse and overdose. Thus, there is a critical need for non-opioid treatments for pain in SCD [10,11]. Marijuana is increasingly being used to help manage chronic pain disorders, including those from cancer. Smoking tobacco, especially in the sickle cell population, increases the risk of acute chest syndrome [12].

Despite the increasing popularity of the use of marijuana and its derivatives, not much has been studied on a large scale concerning its effect on the sickle cell population. In this systematic review, the goal is to ascertain whether marijuana use can help with managing pain in patients with SCD and can be considered as a viable adjunct in hopes of alleviating reliance on opioids and, by extension, reducing hospital admissions.

## Review

Methods 

The Preferred Reporting Items for Systematic Review and Meta-Analysis (PRISMA) 2020 guidelines were employed for conducting this systematic review [13].

Search Sources and Strategy

PubMed, Medline, PubMed Central (PMC), Cochrane Library, and Google Scholar were searched for literature published between 2011-2021.The databases were explored using three search keywords and then combining these results with the Boolean "AND." For the keyword "Marijuana," we used concept identification words: marijuana, weed, ganja, and cannabis. These concept words were joined using the Boolean "OR," and a medical subject heading (MeSH) search strategy was formulated. For the keyword, "Pain," only pain was utilized. For the keyword "Sickle Cell Disease," concept identification words used were sickle cell disease and sickle cell anemia; the Boolean "OR" was again used to combine these two concept identification words. Finally, all MeSH search strategies were compiled using the Boolean "AND," which was then entered into PubMed. The following is the MeSH strategy employed: Marijuana OR Weed OR Ganja OR Cannabis ("Marijuana Use/adverse effects" (Majr) OR "Marijuana Use/drug therapy"(Majr) OR "Marijuana Use/pharmacology" (Majr) OR "Marijuana Use/therapeutic use" (Majr) OR "Marijuana Use/therapy" (Majr) ) AND Pain ("Pain/analysis" (Majr) OR "Pain/drug effects" (Majr) OR "Pain/drug therapy" (Majr) OR "Pain/rehabilitation" (Majr) OR "Pain/therapy" (Majr)) AND Sickle cell disease OR sickle cell anemia ("Anemia, Sickle Cell/drug therapy" (Majr) OR "Anemia, Sickle Cell/rehabilitation" (Majr) OR "Anemia, Sickle Cell/therapy" (Majr))

Eligibility Criteria

Duplicates were screened and removed. Then the remaining papers were screened based on title and abstracts. The full text of the results was then evaluated based on the quality analysis of the paper. Only relevant papers that satisfied >60% of assessment criteria in the quality appraisal were included. Of the articles that were selected, only those published in English during the years 2011-2021 were included. They also had to focus on human subjects, specifically the adult population of >18 years. Papers that focused on animal studies and/or included the pediatric population were excluded. The pediatric population was excluded due to the lack of data on documented use of marijuana in the younger sickle cell population.

Risk Bias Assessment:

The quality of studies included was assessed using the following tools displayed and only articles satisfying >60% of the appraisal parameters were included as seen in Table 1.

**Table 1:** Quality Assessment Using the Preferred Tools SANRA: Scale for the Assessment of Narrative Review Articles; AMSTAR: Assessment of Multiple Systematic Reviews

Type of Study	Tool Used	Number of Studies
Systematic reviews	AMSTAR checklist	1
Randomized controlled trial	Cochrane risk of bias assessment tool	1
Observational studies	Newcastle-Ottawa tool	3
Research papers without clear methods section	SANRA checklist	2

Results

Search Outcome

**Figure 1:**
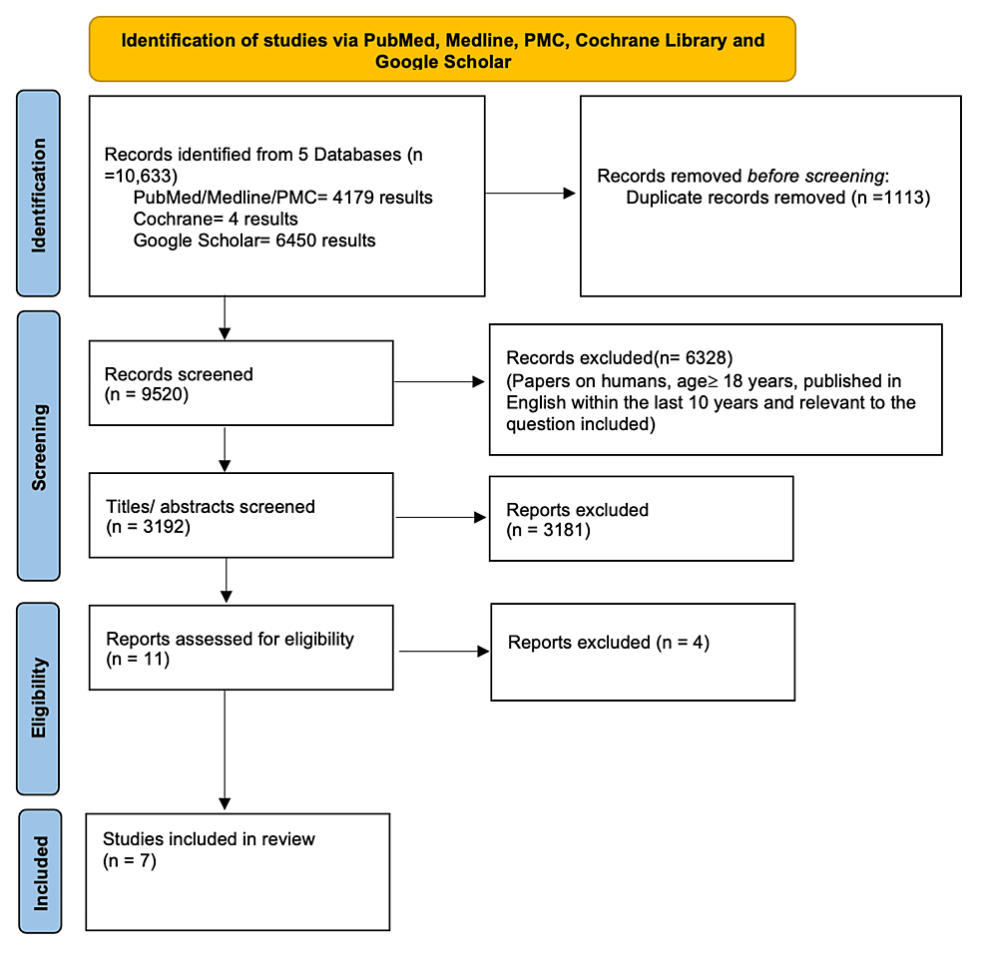
PRISMA Flow Diagram Depicting the Article Selection Process PRISMA: Preferred Reporting Items for Systematic Review and Meta-Analysis; PMC: PubMed Central®

A total of 10,633 papers were identified using the MeSH and keywords through five databases and produced 9,520 results after 1,113 duplicates were removed. Results were preliminarily screened based on the eligibility criteria (inclusion and exclusion) and then on the title and abstract, which produced a total number of 11 articles. After a quality appraisal check, only seven articles were employed. The seven articles included one systematic review, one randomized control trial (RCT), and five observational studies. These articles included the effects of marijuana on patients with sickle cell anemia in terms of pain management and other effects related to cannabis use. The frequency of hospitalizations secondary to painful crises was noted. The PRISMA flow diagram displays the article filtering process as seen in Figure 1.

Actual Results

Our systematic review gathered information from the limited data available on patients with SCD on pain management. This included surveys, retrospective analysis, patient self-reports, and urine screening detection. While all selected articles shared the common objective to study the relationship between marijuana and pain in SCD patients, the number of participants, demographics, and criteria for the studies all differed. The relevant information from each of the seven published papers is summarized in Table 2. In the RCT, 23 patients were studied of which 12 were allocated to cannabis treatment and 11 to placebo [14]. Marijuana was found to be safe to use and significantly improved the participants' mood. Statistically, there was no significant difference between the two groups, but a trend was noted in reducing pain felt in cannabis users compared to the placebo group. In a retrospective study involving 75 participants, it was noted that SCD patients in the first six months after receiving medical marijuana had lower hospital admission rates compared to patients who did not receive it [15]. In contrast, another retrospective study utilizing urine drug screen tests from 40 male and 32 female SCD patients displayed the opposite [16]. The data showed that hospital admissions secondary to vaso-occlusive crises were greater in the cannabis group when compared to the control.

**Table 2:** The Effects of Cannabis on Pain Management in SCD Patients SCD: sickle cell disease; RCT: randomized control trial; AVN: avascular necrosis; VOC: vaso-occlusive crisis

Author and Year of Publication	Purpose of study	Number of Patients/ studies	Type of Study	Conclusion
Curtis et al. 2020 [17]	To examine SCD adults who are daily cannabis users compared to others employing therapeutics using a substantiated patient record of pain, quality of life, and health care usage.	49	Cross-sectional survey	SCD patients who often have worse painful crises were more likely to utilize cannabis daily while lowering the visits to the ER and hospital admissions.
Abrams et al. 2020 [14]	To ascertain the effectiveness of inhaled cannabis compared to an inhaled placebo for chronic pain relief in adults with SCD.	23	RCT	While inhaled cannabis was determined to be safe and more effective in mood interference compared to the placebo, there was no remarkable difference in pain when comparing placebo and cannabis.
Argueta et al. 2020 [18]	To provide relevant data into the risks and benefits of marijuana use in SCD and the various mechanisms of action, outcomes, and categories of cannabis-based treatment strategies.	15 studies	Systematic review	While there is a scarcity of clinical evidence for marijuana effects in SCD, the findings from the papers mentioned lead to the possibility of cannabis analgesic use in managing pain from SCD. Larger RCTs in the context of SCD without the impact of other substances are needed.
Miodownik et al. 2018 [19]	To evaluate the prevalence of marijuana use in an urban population of SCD patients and identify clinical characteristics that may predict marijuana use in this cohort.	78	Survey study	Marijuana use was more likely to be employed as an adjuvant for chronic pain, especially in severe complications of SCD such as AVN, daily pain, and significant opioid use.
Roberts et al. 2018 [20]	To seek more information on how often and for what reasons do patients with SCD use marijuana	57	Survey study	Self-reporting may have skewed results from exaggeration of medical benefits to underreporting of illicit marijuana use.
Ballas 2017 [16]	To ascertain whether SCD patients using cannabis had fewer acute VOCs that required hospitalization	72	Retrospective study	The data displayed that the frequency of VOCs increased with cannabis use in SCD patients than non-users.
Kalu et al. 2016 [21]	To discover the prevalence of marijuana, use among hydroxyurea and non-hydroxyurea users with SCD.	103	Questionnaire-based survey	Based on the data, there was no significant difference in marijuana use in the hydroxyurea and non-hydroxyurea users. It was noted that marijuana use was not entirely dependent on pain control for SCD patients.

Discussion

The Effects of Cannabis on Pain Management in SCD Patients

A cross-sectional survey by Curtis et al. examined SCD adults using cannabis daily contrasted against those who used other therapeutics. Both parties were assessed based on parameters such as quality of life, health care usage, and patient self-reported outcomes [17]. The design of the survey enquired from participants if cannabis was used in the last 30 days. If this was confirmed, it sought further evaluation into other parameters such as frequency of use and reasons for use such as pain, mood, anxiety, sleep, and decreased reliance on other medication [17]. Participants could have chosen multiple options. Of the 49 patients for the past month, 22 confirmed cannabis use with three for monthly use, one used less than one month, eight used it weekly, and 10 reported daily use. Upon examination of the two cohorts, the pain scores were more severe for daily users of marijuana, but they also displayed fewer hospital and ER admissions [17].

Abrams et al. conducted an RCT with 23 participants to determine the effectiveness of inhaled cannabis compared to an inhaled placebo for chronic pain relief in adults with SCD. Similar to the survey conducted by Curtis et al., a pain rating assessment was used to record this data [14,17]. While the sample size in the randomized control trial is almost half of that in the cross-sectional survey, the conclusion derived was that in terms of hydroxyurea use, sex, and pain relief experienced, there were no significant statistical differences [14]. The systematic review by Argueta et al. focused on evidence-based insights on the risks and benefits of marijuana use in SCD, available groups of marijuana-based treatments, the clinical along with the preclinical outcomes, in addition to the mechanism of action of cannabis use. The review focused on 15 studies: one RCT, two cross-sectional surveys, three retrospective studies, one observational study, one qualitative study, one prospective study, one survey study, three case studies, and two questionnaire-based studies. While sample sizes, demographics, and other parameters differed for almost all these studies, one commonality was that further data is needed as well as long term studies ideally with less interference from other drugs to make decisive proposals on whether marijuana use is efficacious for pain relief in SCD or not [18].

Miodownik et al. surveyed 78 patients from 2017 to 2018 to determine the reasons that may contribute to SCD patients using marijuana, the prevalence of this occurring in urban populations in the United States, as well as the clinical characteristics that may lead to this. The survey was conducted at outpatient centers focused on caring for adult patients with SCD [19]. The data was gathered from patient self-reporting and answering of questions concerning marijuana use, disease complications, and demographics. The results, which compared marijuana users with non-users from the survey, revealed that marijuana users were likely to be male compared to non-users, to be prescribed opioids (93% vs. 65%), to have avascular necrosis (47% vs. 17%), and to report daily pain (40% vs. 13%) [19]. In contrast to the study done by Curtis et al., the SCD patients from this survey who used marijuana were more prone to being diagnosed with avascular necrosis, and they reported more ED visits, as well as an increase in the frequency of daily pain experienced [19].

Roberts et al. sought data on reasons why SCD patients would use marijuana in the United States even though this usage may not have been legal in the state in which they resided. A survey study was carried out in an urban medical center, which catered specifically to SCD adults [20]. A self-report survey about marijuana use over two years was created, validated, and eventually administered to 57 patients. Among those who used marijuana, 92% reported that their purpose to use the substance was driven by their need for pain relief [20]. In fact, 79% of the users agreed that due to the pain relief experienced with cannabis use, their need to rely on pain medications was reduced [20]. Based on the findings of this survey, Roberts et al. concluded that there is precedent to delve more into the therapeutic effects of marijuana and including SCD as a condition that qualifies for medical marijuana usage [20].

In 2017, Ballas was determined to discover whether SCD patients using marijuana had less acute VOCs that required hospitalization. A retrospective study involving 72 adult African American patients from a sickle cell center from 1994 through 2009 was employed [16]. The goal of this study focused mainly on documenting clinical features experienced by SCD patients using marijuana. This was ascertained by screening random urine drug tests and comparing those that showed no cannabis to those that did against the frequency of VOCs. Key findings that emerged from this study were that pain relief was the number one reason for regularly smoking marijuana in all the patients who tested positive for the substance [16]. Despite this, the results showed the opposite. Ballas reported that patients identified as cannabinoid users presented to the hospital for treatment of VOCs more frequently as compared to the control group [16]. Interestingly, in addition to increased hospitalizations, there was a noted decline in clinic visits, the reason for which is unknown [16]. 

Kalu et al. formulated a self-administered anonymous questionnaire that was distributed amongst 103 SCD patients. The purpose of this study was to determine how often marijuana was used amongst SCD patients, with a focus on those who did and did not utilize hydroxyurea as part of their treatment plan [21]. In contrast to Kalu et al.'s study, more women (51%) than men (49%) were participants in Ballas' study [16,21}. The main reason for marijuana usage among participants, especially those who used it within the past year (30%), was to alleviate symptoms associated with SCD [21]. Despite this, sickle cell-related pain was not effectively treated with marijuana when compared to hydroxyurea, according to 80% of participants. Additionally, there was no significant variance in the marijuana usage among hydroxyurea and non-hydroxyurea users compared to the general population [21]. Kalu et al. had the most participants out of all the literature analyzed for this systematic review, and 103 patients were surveyed to ascertain the prevalence of cannabis use in hydroxyurea and non-hydroxyurea users with SCD [21].

The Effects of Cannabis on Mood in SCD Patients

While marijuana use was being observed for its effects on pain relief, some of the literature published noted additional adverse effects on the participants. In the RCT conducted by Abrams et al., there was no difference in pain rating between the placebo and marijuana participants [14]. However, there was an interference with mood, which was statistically significant when compared from day one to day five in SCD patients who were experiencing chronic pain [14]. Curtis et al., in their cross-sectional study, also came across reasons as to why SCD participants opted to employ marijuana. Based on the patients' self-reports, besides pain, the most common reasons noted were anxiety and mood [14]. However, the impact of cannabis on mood and anxiety was insignificant when compared among cannabis users and non-users and then contrasted to the reference population [14]. Roberts et al. also conducted a study involving 57 SCD patients and noted their response to the survey administered. Once again, while pain (92%) was the major rationale for utilizing cannabis, anxiety (72%) and mood (67%) were also listed [20]. The paper stops short of any further information on mood and anxiety as its focus was mainly on pain. However, it is something that can be investigated for future research. Similarly, to Roberts et al., the main motive for marijuana use was pain, stress, anxiety, concentration, and mood. Reasons for cannabis use in SCD patients other than for pain relief need to be explored more for any significant correlation between use and effect [20,21].

Limitations

This systematic review, which consisted of seven published pieces of literature, has limitations. One such limitation was that only one RCT was sourced from our five databases. This could have been partly due to our eligibility criteria, which focused on literature published in English within the past 10 years with a focus on adults greater than 18 years of age. As a result, several papers may have been excluded simply because they did not meet the inclusion/exclusion criteria. There were also differences in the number of subjects between papers such as Kalu et al., which had a sample size of 103 participants, compared to Roberts et al., which had only 57 patients. The individual limitation of each study regarding study length, sample size, and drug effects made it difficult to facilitate adequate comparisons.

## Conclusions

We aimed to determine whether marijuana can be used to relieve pain in SCD patients and, therefore, by extension, can be successfully employed as an adjunct in the future to reduce the frequent and often dependent use of opioids. However, based on the results of the literature gathered, there seems to be a split decision. Not many clinical trials were found to substantially address whether cannabis use significantly impacted improving pain relief in SCD patients. While the information gathered from the different surveys and studies did provide some insight, they contradicted each other in terms of findings. Therefore, at this point, a definitive conclusion cannot be made. Ideally, new research is necessary to further address this topic with a focus on the long-term effects of cannabis use and objectively assessing pain relief utilizing records on hospital/ED visits for more than two years.
